# Performance of Ceramic-Metal Composites as Potential Tool Materials for Friction Stir Welding of Aluminium, Copper and Stainless Steel

**DOI:** 10.3390/ma13081994

**Published:** 2020-04-24

**Authors:** Mart Kolnes, Jakob Kübarsepp, Fjodor Sergejev, Märt Kolnes, Marek Tarraste, Mart Viljus

**Affiliations:** Department of Mechanical and Industrial Engineering, Tallinn University of Technology, Ehitajate Tee 5, 19086 Tallinn, Estonia; jakob.kubarsepp@taltech.ee (J.K.); fjodor.sergejev@taltech.ee (F.S.); mart.kolnes1@taltech.ee (M.K.); marek.tarraste@taltech.ee (M.T.); mart.viljus@taltech.ee (M.V.)

**Keywords:** cermet, hardmetal, friction stir welding, diffusion-controlled wear, adhesive wear

## Abstract

The aim of the research was to disclose the performance of ceramic-metal composites, in particular TiC-based cermets and WC-Co hardmetals, as tool materials for friction stir welding (FSW) of aluminium alloys, stainless steels and copper. The model tests were used to study the wear of tools during cutting of metallic workpiece materials. The primary focus was on the performance and degradation mechanism of tool materials during testing under conditions simulating the FSW process, in particular the welding process temperature. Carbide composites were produced using a common press-and-sinter powder metallurgy technique. The model tests were performed on a universal lathe at the cutting speeds enabling cutting temperatures comparable the temperatures of the FSW of aluminium alloys, stainless steels and pure copper. The wear rate of tools was evaluated as the shortening of the length of the cutting tool nose tip and reaction diffusion tests were performed for better understanding of the diffusion-controlled processes during tool degradation (wear). It was concluded that cermets, in particular TiC-NiMo with 75–80 wt.% TiC, show the highest performance in tests with counterparts from aluminium alloy and austenitic stainless steel. On the other hand, in the model tests with copper workpiece, WC-Co hardmetals, in particular composites with 90–94 wt.% WC, outperform most of TiC-based cermet, including TiC-NiMo. Tools from ceramic-metal composites wear most commonly by mechanisms based on adhesion and diffusion.

## 1. Introduction

Hardmetals (WC-based ceramic-metal composites) are extensively used under demanding wear resistance and high stiffness, e.g., in metal cutting and forming tools. The excellent wear resistance exhibited by the hardmetals is due to their combination of high hardness, wear resistance and moderate fracture toughness [1]. Cobalt is widely used as the binder metal because of its good wetting behavior and good mechanical properties. Hardmetals can be used as a tool material for friction stir welding (FSW), especially FSW of high melting point alloys [2,3] or metals with reinforcing particulates [4].

By their definition, cermets consist of ceramic particles bonded with a metal matrix, except for hardmetals that are WC-based [5]. Cermets exhibit high hardness and wear resistance at high cutting rates, as compared to WC-Co hardmetals. The most used metallic binder systems of TiC- and Ti(C, N)- based cermets are Ni alloys. However, Fe alloys have advantages over Ni and Co as metallic binders, such as potential to heat treatment, high strength and low cost. Therefore, research of completely or partially Ni- and Co-free metal composites has been intensified markedly during the last two decades. In general, TiC cermets are applied where low density, high wear resistance or high temperature oxidation resistance is needed. In particular, TiC-cermets with a steel binder have demonstrated their superiority over ordinary WC-hardmetals in metalforming operations owing to their excellent adhesive wear resistance and low fatigue sensitivity [6].

**Figure 1:**
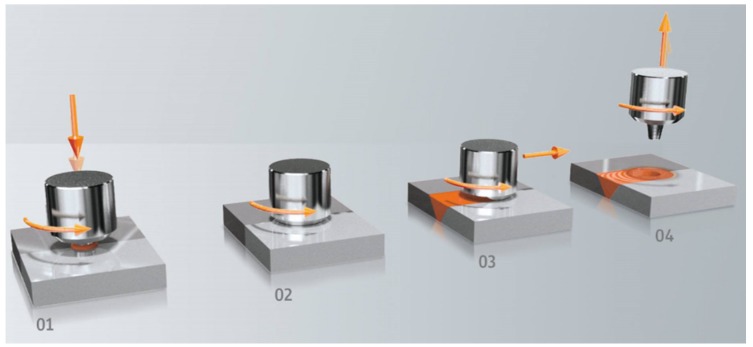
Process steps: (1) approach and plunge; (2) dwell for heating; (3) welding; (4) end of welding.

Friction stir welding (FSW) was invented at The Welding Institute (TWI) of the United Kingdom in 1991 as a solid-state joining technique, initially applied to aluminum alloys [7]. FSW is a solid-state joining process that uses a non-consumable rotating tool with a specially designed geometry to join materials without melting them. Heating is created within the workpiece by friction between the rotating tool and welded material. The localized heating softens the material around the pin and while the tool is traversed along the joint line, it mechanically intermixes the two welded materials (see Figure 1). To avoid defects and welding tool fracture, the process temperature should not exceed 0.7 times of the melting workpiece material. The recommended temperatures range for aluminum alloys, stainless steel and copper 425–500 °C [8], 1000 °C [9] and 700–750 °C [10], respectively.

The solid-state nature of FSW leads to pronounced advantages over traditional fusion welding methods. One of them is the fully recrystallized, fine-grained microstructure created in the nugget by the deformation at elevated temperature. Secondly, there are no conduction problems associated with cooling from the liquid phase, such as solidification cracking, liquation cracking, porosity, and alloying element loss [11]. In addition, FSW is an emergent green technology due to its environmental friendliness and energy efficiency [12]. In comparison with other welding methods, FSW consumes considerably less energy, no shielded gas or filler materials are needed, and no toxic fumes are created during welding—FSW has low environmental impact.

Although originally FSW was invented for aluminium alloys, today it is possible to weld a wide range of metals including copper alloys, magnesium alloys, titanium alloys, stainless steels and even polymers [13]. In this work, we focused on aluminium, copper and stainless steel, because of their mediocre weldability if compared to the low-carbon steel fusion welding [14]. As a solid-state welding process, FSW can avoid the welding defects associated with fusion welding processes [4]. Each material to be welded requires a special approach for selecting the appropriate tool material. Weld quality and tool wear are two important considerations in the tool material selection. Rai et al. give the best understanding of the tool materials used for aluminum alloys and stainless steels FSW [15]. Chromium-molybdenum hot work air hardening tool steel X40CrMoV (AISI H13) and polycrystalline cubic boron nitride (pcBN) are the most widely used tool materials for aluminium alloys and stainless steels, respectively. Arulmoni et al. presents a good review of the tool materials used for friction stir processing (FSP) and FSW of copper [16]. Tool steel AISI H13 is the most common choice for the FSP/FSW of copper. Table 1 presents the tool materials widely used to weld aluminium alloys, copper and stainless steels.

The tools have to withstand severe wear conditions during the FSW process. The degradation (wear) mechanism is complicated and depends on the interaction between the workpiece and the tool material, welding parameters and tool geometry. Studies on the tool wear mechanism in FSW are scarce; at the same time, adhesion, abrasion and diffusion-controlled wear are the expected wear mechanisms [15]. Wear through abrasion is substantial in the presence of a harder second phase in the workpiece material such as in aluminium matrix composites [4].

According to authors’ knowledge, no comparative studies have been performed for revealing performance and degradation mechanism of ceramic-metal composites, as potential tool material, for FSW of different metals with substantially different welding temperatures. The present paper aims to demonstrate the feasibility of ceramic-metal composites use, in particular TiC-based cermets and WC-Co hardmetals, as tool materials for FSW of aluminium alloys, copper and stainless steels. The property requirements for cutting (machining) and FSW tools are quite similar [15]. Therefore, we used the model tests to study the wear of tools during workpiece material cutting. The main focus is on the performance and degradation mechanism of tool materials in the model tests simulating the wear of FSW tools.

## 2. Materials and Methods

### 2.1. Material Specifications

According to [17], the characteristics that have to be considered in selection of tool material for FSP/FSW include high compressive yield strength at elevated temperatures, no harmful reaction with workpiece material, resistance to wear (degradation), good strength and creep resistance, affordable cost etc. Ceramic-metal composites can be considered as significant FSW tool material candidates as they have high hardness, wear resistance and good mechanical properties at ambient and elevated temperatures [1].

The wear of tools made from carbide composites (WC-Co hardmetals and TiC-based cermets) during the model cutting tests of workpiece materials (Al alloy, Cu and austenitic stainless steel) was studied. The chemical composition and characteristics of starting powders for preparation of WC- and TiC- based ceramic-metal composites are shown in Table 2. The initial (starting) chemical composition, carbide content and average particle size in the sintered carbide composites and their mechanical properties are shown in Table 3. The content of tungsten carbide in the WC-Co hardmetals was 85–94 wt.% (76.4–90.0 vol.%).

In our study, TiC-based cermets were included taking into account raw materials supply, environmental safety as well as healthcare concerns related to the utilization of W and Co in hardmetals [18,19]. The most widely used metallic binder system of TiC- and Ti(C, N)- based cermets are NiMo alloys [5]. While Fe-alloys have additional advantages over Co and Ni such as low cost, potential to heat treatment and high strength, also Fe-alloy bonded TiC-FeCr and TiC-FeNi cermets were under investigation. The content of titanium carbide in TiC-based cermets was 70–80 wt.%, ensuring the fraction comparable that of the hardmetals carbide fraction of 79.3–92.1 vol.% (see Table 3).

**Figure 2:**
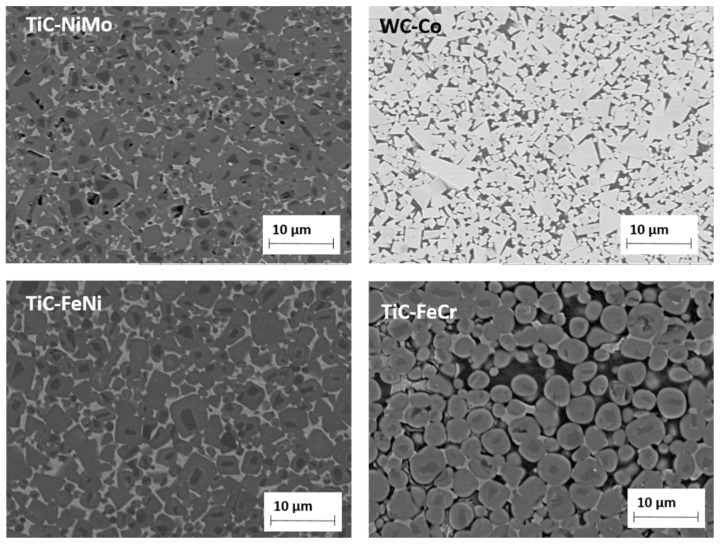
SEM micrographs of 75TiC-NiMo, 85WC-Co, 70TiC-FeNi and 70TiC-FeCr.

The carbide volume content was calculated taking into account the dissolution of Mo in TiC and the formation of secondary Cr-based carbides in TiC-NiMo and TiC-FeCr cermets, respectively [20]. The microstructures of selected carbide composites are shown in Figure 2. All the composites were produced using conventional powder metallurgy technology routes for hardmetals and cermets. Powder mixtures for starting powders were prepared using conventional ball mill with WC-Co lining and balls for duration of 72 h at the ball to the powder weight ratio of 10:1. Isopropyl alcohol was used as a milling liquid. The WC-Co contamination from milling was approximately 5 wt.% in case of TiC-based cermets. Tungsten carbide dissolves in titanium carbide and cobalt in metallic binder. Milled powders were mixed with 3 wt.% paraffin, granulated, dried and compacted into green bodies with 100 MPa of uniaxial pressure. Pressureless liquid phase sintering in vacuum (0.1–0.5 mbar) during 30 min was carried out to obtain specimens for the mechanical and model (cutting) tests. The optimal sintering temperatures of 1450–1500 °C (depending on the composition) for achieving near-full densification (low porosity) and maximal mechanical characteristics were used. Heating rate up to the sintering temperature was 10 °C min^−1^, followed by dwelling at the final temperature and cooling with furnace. The porosity of the consolidated ceramic-metallic composites was under 0.5 vol.%. The porosity was evaluated by measuring the surface area of pores on the optical image of the microstructure at the magnification of 200 times. Optical microscope Axiovert25 (Carl Zeiss AG, Oberkochen, Germany) and software Buehler Omnimet were used. The chemical composition of the counterpart materials for the model tests is shown in Table 4.

### 2.2. Experimental Details

**Figure 3:**
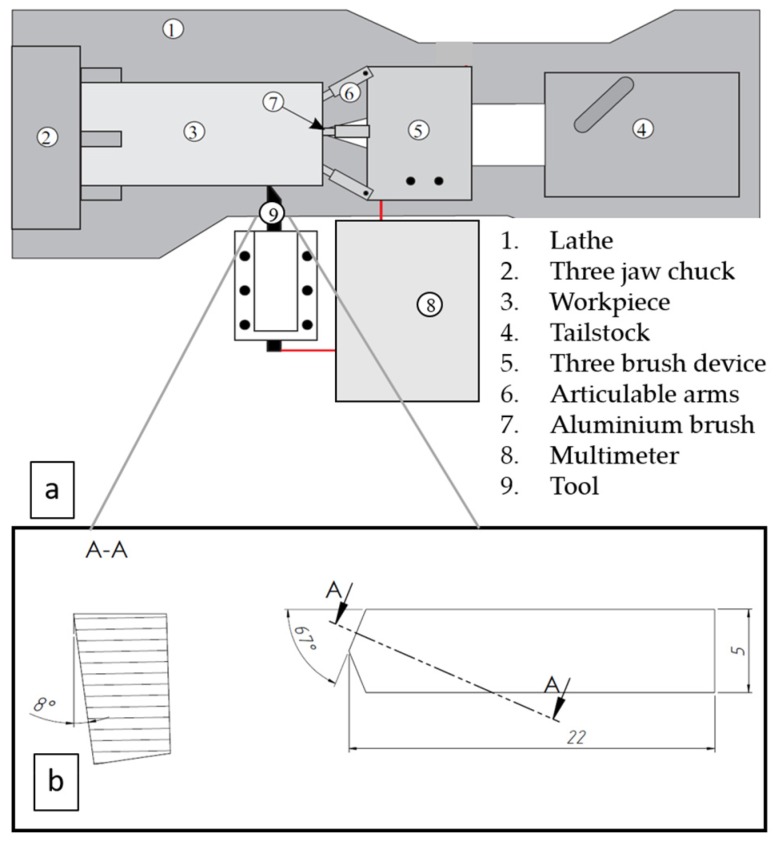
Schematic of the tool-workpiece thermocouple system (**a**) and cutting tool geometry (**b**).

To evaluate the wear performance of different carbide composites during the FSW of aluminium alloy, copper and stainless steel, the model tests were performed on a universal lathe (see Figure 3a), using cutting tools from carbide composites with specific geometry (see Figure 3b). Tools were grounded to achieve a nose angle of 134°, side cutting edge angle of 23° and side relief angle of 8°. The set of three specimens were produced for every tested material, to assess the reliability and the reproducibility of experimental results. The investigated material bars were machined at high speed, up to 630 m/min, 470 m/min and 280 m/min to achieve the cutting zone temperature approximately similar to that of the welding temperature for aluminium alloy (400 °C), copper (600 °C) and stainless steel (1000 °C), respectively. The feed rate and the depth of the cut were kept constant during testing at 0.39 mm/rev and 0.125 mm, respectively. Common FSW temperatures for Al-alloys (~400 °C) and stainless steel (~1000 °C) were achieved at described cutting speeds, while for copper temperature of 600 °C, which is somewhat below regular FSW temperature (700–750 °C), was achieved as maximum.

**Figure 4:**
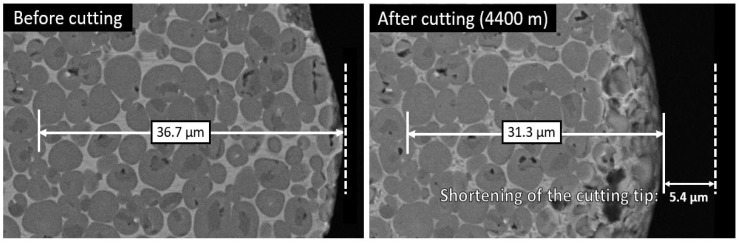
The measurement of the shortening of 70TiC-FeCr tool on the top surface of cutting nose tip after cutting aluminium alloy (cutting distance 4400 m).

To measure the cutting temperature in the cutting point, a tool-workpiece thermocouple method was used for all tested materials [21]. Detailed calibration, temperature measurement and cutting wear test parameters were described in [22]. The wear rate of the tool was determined as the shortening in the length of the cutting tool nose tip of the model test specimens. Shortening of the cutting nose tip was measured on the top surface of the tool in the case of aluminium alloy and copper as workpiece materials, using the images made from the top surface of the cutting tool by Hitachi TM 1000 Tabletop scanning electron microscope (Hitachi High-Tech, Krefeld, Germany) (see Figure 4). In the model tests with stainless steel, the height of the wear pattern was measured on the front surface of the tool because the tool wear rate was significantly more intensive. These measurement results were recalculated to the shortening of the cutting nose tip on the top surface for better comparison.

In the present research reaction diffusion tests were performed for better understanding of the diffusion controlled processes during tool degradation The reaction diffusion of tool material is one of the important reasons of FSW tool degradation by intergrain fracture during working of aluminium alloys, especially if taking into account the acceleration of diffusion by temperature and contact stresses developed in the FSW. Fragments of the FSW tool material are deformed and detached from the FSW tool by fracture along the embrittled grain boundaries under the shear stress developed on the surface of the tool during FSW [23]. Diffusion of workpiece material elements to tool is especial concern in FSW/FSP of high-melting point metals. As an example FSW tool wear by rapid-rate diffusion of elements from Ti- alloy into W-Re alloy tool causing degradation and wear by subsurface fracture has been reported [24]. Similar processes are significant considering FSW of steels and copper characterized by much higher working temperatures and contact stresses if compared to aluminium alloys.

It is known, that during the FSW of aluminium alloys FSW tool is covered by tribological deposits [23]. Similarly, during model cutting tests of aluminium alloy ceramic-metal tools were covered by strongly adhered layers of the workpiece material [22]. Such layers enable analysis of diffusion processes in the contact region of tool-workpiece. Unfortunately, the formation of such tribological layers was not evident during the model tests using stainless steel and copper as workpiece materials. Therefore, the special test samples were prepared for all tool and workpiece materials groups. Ceramic-metal composites with the highest binder fraction from each tested tool material group were selected (85WC-Co, 70TiC-NiMo, 70TiC-FeCr, 70TiC-FeNi) for achieving deeper diffusion of elements from workpiece materials into tool. The test samples for studying diffusion-controlled processes were prepared using spark plasma co-consolidation of sintered carbide composite specimens and workpiece material or material powder with similar chemical composition to obtain a permanent bond between the tool material and the metal (Al-alloy, copper, stainless steel). Reaction diffusion tests were performed by heating the test samples up to approximately the same temperature as in the model tests, followed by 4 h dwelling at that temperature in vacuum furnace. Such heating time is relatively short in comparison with FSW tool life during welding of aluminium alloys and copper. To understand the distribution of chemical elements in the microstructure in the tool-workpiece contact region, the scanning electron microscope (Zeiss EVO MA15, Oberkochen, Germany) equipped with energy-dispersive X-ray spectroscopy system INCA (Oxford Instruments, Wycombe, UK) was used.

Hardness and fracture toughness of sintered composites were determined using ground test specimens of 5 mm × 5 mm × 17 mm. Hardness (HV30) was measured with a hardness tester Indentec 5030KV (Indentec Hardness Testing Machines Limited, West Midlands, UK). Toughness was evaluated with the indentation method (Palmqvist method) using equation described in [25].

## 3. Results

**Figure 5:**
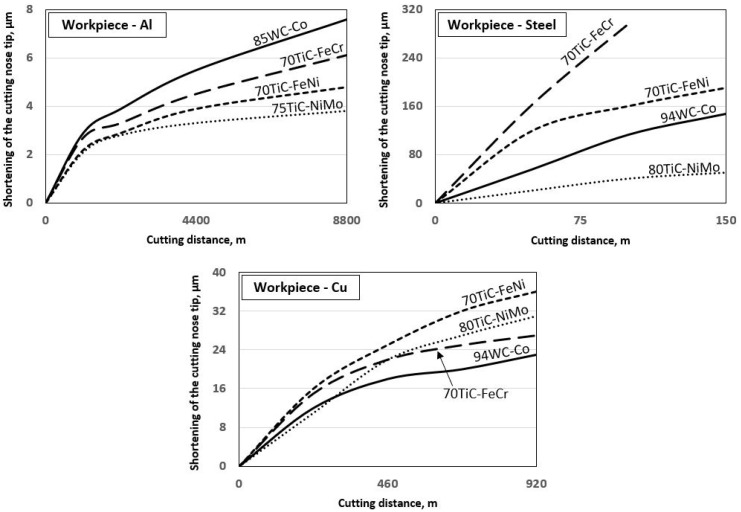
Wear of ceramic-metal composites vs. cutting distance in the case of all workpiece materials at work temperatures.

**Figure 6:**
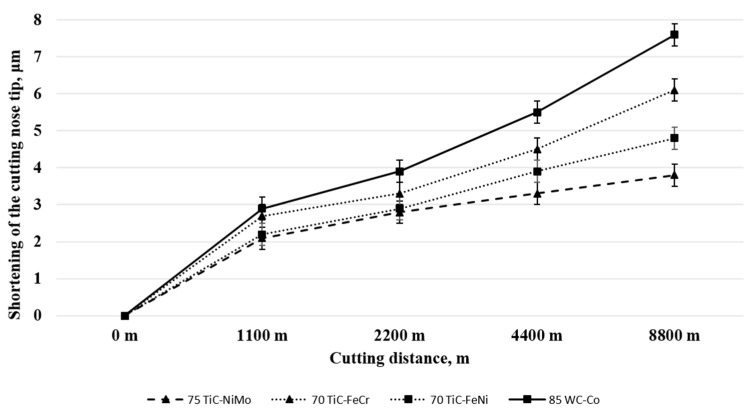
Wear of ceramic-metal composites vs. cutting distance in the case of aluminium alloy at 400 °C.

**Figure 7:**
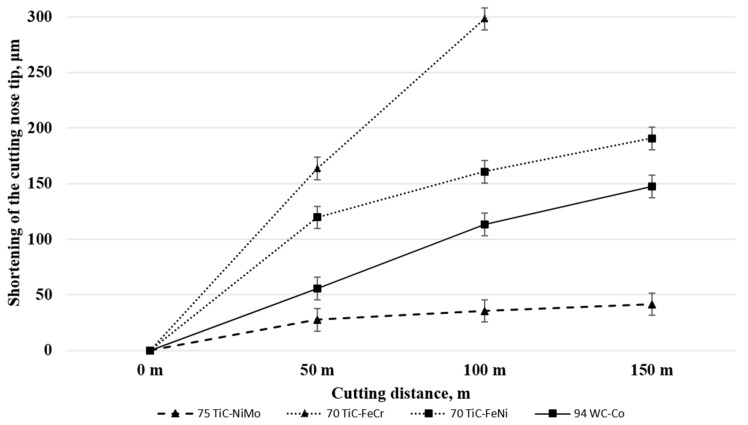
Wear of tested ceramic-metal composites vs. cutting distance in the case of stainless steel at 1000 °C.

Table 5 and Figure 5 presents tool materials wear rate (shortening of the cutting tool nose tip) in model tests with different workpiece materials. (Figure 5, Figure 6 and Figure 7 for aluminium alloy, stainless steel and copper, respectively). Maximal cutting distances were 8800 m, 150 m and 920 m in case of aluminium alloy, stainless steel and copper, respectively. Although aluminium alloy is the softest of workpiece materials the cutting process proved to be unstable and tools were subjected to severe dynamic loads. Therefore, the hardmetal and cermets with high binder fraction and fracture toughness were used in tests with workpiece material from aluminium alloy.

### 3.1. Working of Aluminium Alloy

Figure 6 and Table 5 demonstrate the wear performance plotted against the cutting distance of the WC-Co hardmetal and TiC-based cermets. Wear of ceramic-metal tools during model tests with workpiece from aluminium alloy at 400 °C is very small even at considerable cutting distances. For that reason, selected composites (5) from Table 3 were tested.

WC-Co hardmetal 85WC-Co demonstrated the highest wear rate during testing. This ceramic-metal composite showed also the highest wear gain during an increase of the cutting distance (compare wear rate increase angles of different composites in Figure 6). All TiC-based cermets showed an advantage over the hardmetal. However, cermets with NiMo binder are at advantage over composites with Fe-alloy binders (FeCr and FeNi).

### 3.2. Working of Stainless Steel

Figure 7 and Table 5 demonstrate wear (shortening of the cutting tool nose tip) plotted against the cutting distance of ceramic-based composites of different composition and carbide fraction. For clarity, Figure 7 shows testing results only for the most wear resistant grades of WC-Co hardmetals and TiC-NiMo cermets in Table 5. Unlike the model test results with aluminium alloy workpiece (at 400 °C), the wear of cermet and hardmetal tools was pronounced during cutting of stainless steel (at 1000 °C) even at comparatively short cutting distances (see Figure 5). Increase in the volume fraction of the carbide phase and hardness in the composites enhances wear performance of WC-Co hardmetals and TiC-NiMo cermets. Regardless of high chromium content, ensuring enhanced resistance to oxidation, FeCr alloy bonded cermet showed the highest wear rate as well as wear gain during the increase of the cutting distance. An alternative Fe-alloy bonded 70TiC-FeNi cermet exhibited better wear performance at the level of WC-Co hardmetals. TiC-NiMo cermets with 75–80 wt.% TiC demonstrated superior wear performance in such conditions. Such results prove different wear mechanisms during testing of aluminium alloy workpiece and stainless steel at substantially different working temperatures.

### 3.3. Working of Copper

**Figure 8:**
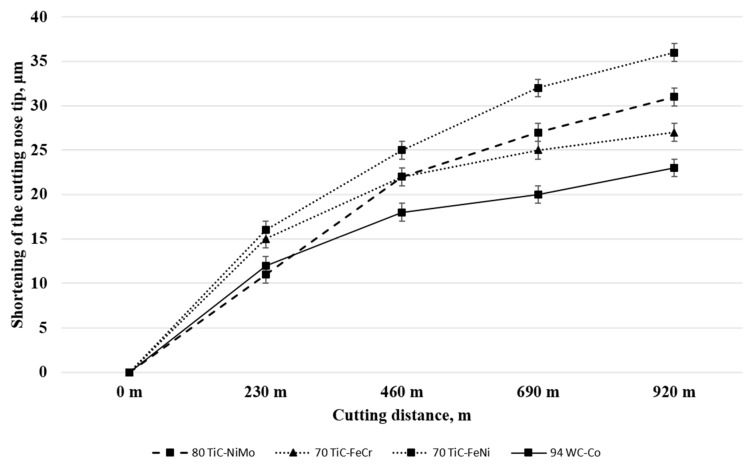
Wear of ceramic-metal composites vs. cutting distance in the case of copper at 600 °C.

Figure 8 and Table 5 show wear performance plotted against the cutting distance of WC- and TiC- based ceramic-metal composites of different composition and fraction of carbides. For clarity, Figure 8 presents the testing results only for the most wear resistant grades of WC-Co hardmetals and TiC-NiMo cermets. Increase in the carbide (WC or TiC) fraction improves the wear performance of both WC-Co hardmetals and TiC-NiMo cermets (see Table 5). As different from the model tests with workpieces from aluminium alloy and stainless steel, WC-Co hardmetals with 90–94 wt.% WC demonstrated the best performance. It should be pointed out that 70TiC-FeCr cermet also showed high wear resistance, which, however, demonstrated the lowest performance during working of stainless steel (see Figure 5). Regardless of substantial performance of TiC-NiMo cermets in the model tests with Al alloy and stainless steel, NiMo-alloy bonded cermets compare unfavourably with WC-Co hardmetals when the counterpart is copper. These results demonstrate different wear mechanisms during testing (cutting) of workpieces from different metals at substantially different temperatures and contact stresses.

## 4. Discussion

The tools have to bear severe wear during the FSW process and during the model cutting tests used in our research. Abrasion, adhesion as well as diffusion-controlled wear are the expected wear mechanisms. Previous research [26,27,28] has shown that at a similar volume fraction of carbides the TiC-based cermets, independent of their binder composition and structure in abrasive-erosion and in the abrasive wear conditions, are at a disadvantage in relation to the WC-based hardmetals. However, cermets with a steel binder are at an advantage over cermets with a NiMo-binder (at room temperature). The performance of carbide composites in abrasion conditions is controlled primarily by the stiffness of the composite—its resistance to elastic (evaluated by the modulus of elasticity) and plastic (evaluated by proof stress) straining and depends primarily on the properties of carbide phase (WC vs. TiC). Prognosis of abrasive wear performance based on hardness may lead to pronounced mistakes when composites based on different ceramic phase are considered [26,27].

Adhesive wear performance (unlike performance in abrasion conditions) of cermets may be comparable to that of WC-based composites. It is controlled primarily by composite resistance to local plastic straining featured by the proof stress, depending both on the properties of metallic and ceramic phases. Similar to abrasion, inconclusive influence of hardness on the adhesive wear performance of ceramic-metal composites with substantially different composition must be highlighted. However, an increase in ceramic fraction and hardness in composites based on similar carbides and metallic binder results in the improvement of the wear performance [26,28].

Tool degradation under FSW conditions and during the model cutting tests is increased at elevated temperatures, by the reactions of the tools with the workpiece and with the oxygen-containing atmosphere. Therefore, reactivity of the tool material also accounts for severe wear during working at high temperatures. Diffusion controlled chemical reactions determine the nature of the interface formed between the tool and the workpiece. Tool material properties will alter at high temperatures, leading to a higher likelihood of tool degradation or even failure.

### 4.1. Working of Aluminium Alloy

Figure 6 and Table 5 demonstrate that TiC- based cermets with Ni- and Fe- alloy binders are at an advantage over WC-Co hardmetal. It may be explained to a considerable extent by the higher carbide fraction (see Table 3) as well as hardness of cermets. 94WC-Co hardmetal with substantially higher carbide fraction and hardness (and the lowest fracture toughness) suffered structural failure after comparatively short cutting distances.

During working (cutting) of plastic metals with the fractional content of hard second phases, tools fail most commonly by mechanisms based on adhesion and diffusion. Adhesion as the key wear mechanism is the reason why result of TiC-based cermets, in particular TiC-NiMo composites, demonstrate superiority over the WC-Co hardmetal during working of aluminium alloy. Adhesion as the wear mechanism is confirmed by strongly adherent workpiece metal deposits (layers) on the rake face of ceramic-metal tools. Other researchers addressing the wear mechanism of tools during FSW of Al- [23] and Ti- alloys [29] have made similar observations. Also, Al diffusion has been shown to be a degradation factor in the tools used for FSW of aluminium [23]. These diffusions-controlled reactions are possible due to the combinations of both local high thermal and high mechanical loading effects on the tool. It may lead to the formation of hard and brittle intermetallic compounds in heat affected zones of Co-, Ni- and Fe- based binders of ceramic-metal composite tools. The embrittlement created at the grain boundaries of a tool material may increase the likelihood of degradation under severe stresses that the tool surfaces are exposed to.

**Figure 9:**
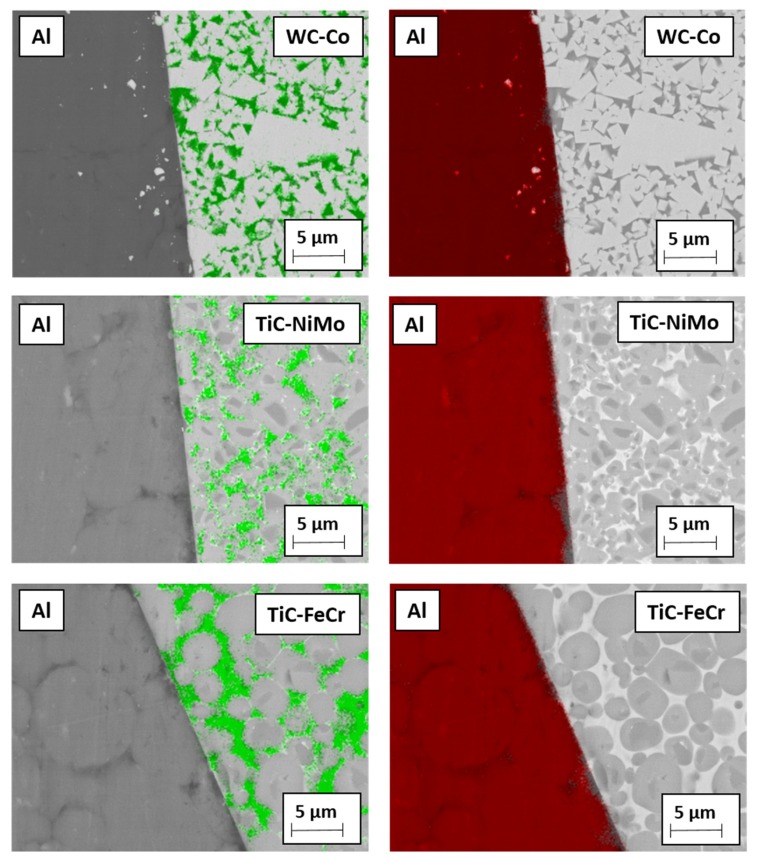
EDS mapping of permanent bond between tool materials and aluminium alloy heating in vacuum during 4 h at 400 °C: green—metallic binder (Co, Ni, Fe), red—aluminium.

**Figure 10:**
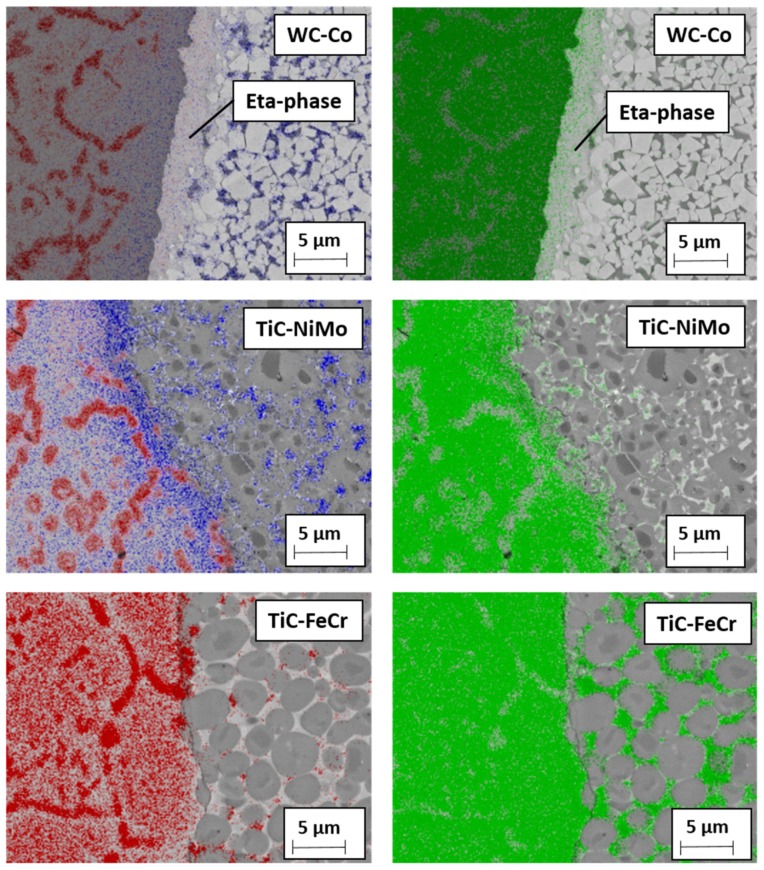
EDS mapping of permanent bond between tool materials and stainless steel after heating in vacuum during 4 h at 1000 °C: red—chromium, green—iron, blue—metallic binder (Co, Ni).

**Figure 11:**
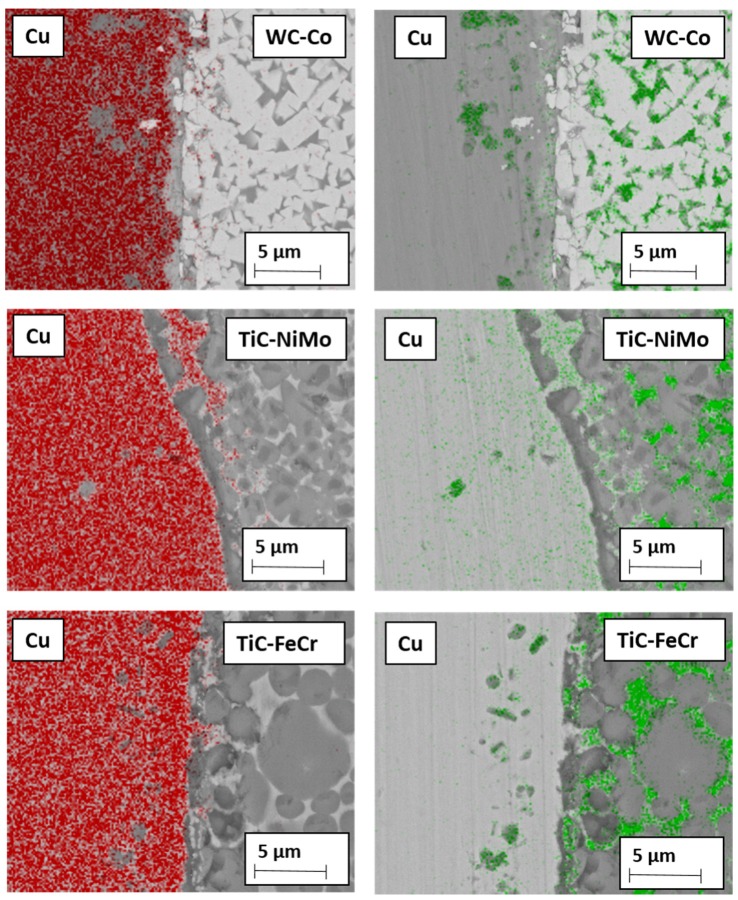
EDS mapping of permanent bond between tool materials and copper after heating in vacuum during 4 h at 600 °C: red—copper, green—metallic binder (Co, Fe, Ni).

Figure 9 demonstrates the EDS mapping of Co-, Ni- and Fe- based binders in contact with Al-based workpiece material after heat treatment of 4 h at 400 °C. Diffusion of aluminium into ceramic-metal composites metallic phase is quite marginal as compared to the diffusion of stainless steel components and copper at 1000 °C and 600 °C (see Figure 10 and Figure 11), respectively. Detailed study described in [22] based on the EDS line scan of the contact region “tool-aluminium alloy” has demonstrated that the diffusion of Al was the highest into the WC-Co hardmetal both in depth and intensity (content) while the lowest into the TiC-NiMo cermet. The highest wear gain during an increase of the cutting distance of the WC-Co hardmetal can be explained by the formation of hard and brittle intermetallic phases in the contact region of the tool-workpiece, whereas their amount increases with an increase in the aluminium content.

### 4.2. Working of Stainless Steel

Figure 7 and Table 5 show that TiC-NiMo cermets with 75–80 wt.% TiC outperform 94WC-6Co hardmetal with the highest hardness (at room temperatures) among ceramic-metal composites considered. TiC-based cermets with Fe-alloy binders are at a disadvantage in relation to all ceramic- metal composites under investigation.

Ni–based alloys can withstand severe operating conditions involving high temperature and corrosion environments. Such alloys with excellent elevated temperature properties, in particular NiMo alloys, are used at the highest homologous temperature if compared to any common metallic alloy systems [30,31]. The Ni-based binders in cermets can decidedly handle the heat generated during FSW and the model cutting process better than cobalt in WC-Co hardmetal and Fe-based alloy in the TiC-FeNi and TiC-FeCr cermets.

Experimental investigation of WC-based tool materials wear during the FSW of stainless steel has shown that the wear at a pin root and at the bottom face of the pin is attributed mainly to diffusion, adhesion and attrition mechanisms [3]. Hot adhesion is one of the wear mechanisms behind the result that TiC-NiMo cermets demonstrate superiority over abrasive wear resistant WC-Co hardmetals during working of stainless steel.

During cutting of stainless steel, tool materials are exposed to high temperatures affecting remarkably the mechanism of wear. The presence of Fe and Cr in stainless steel strongly influences the dissolution of carbide particles present in the WC-Co hardmetal as well as in TiC-based cermet tool materials. Dissolution of WC leads to the formation of carbon deficient forms of tungsten carbide W_2_C, Co*_x_*W*_y_*C compounds (eta phase) and M_23_C_6_ phases [32]. During welding at high temperatures, the Fe present in the stainless steel also diffuses into the tool and forms a solid solution with cobalt [3]. The carbon liberated during the dissolution of WC dissolves in the Co-Fe solid solution and its solubility increases as the amount of Fe in the solid solution increases. Brittle metallic carbide compounds are easily removed by mechanical action, giving rise to craters on the surface of the tool [3]. These diffusion-controlled processes—formation of carbon deficient forms of tungsten carbide and metallic carbides in Co binder—remain relevant wear processes during FSW and during cutting in the model wear tests with the WC-Co tool.

Figure 10 demonstrates the EDS mapping of the WC-Co hardmetal and TiC-based cermets in contact with stainless steel after heat treatment 4 h at 1000 °C. The figure shows the formation of brittle carbon deficient tungsten carbide (eta phase) interaction layer at the interface between the cemented carbide and steel. High stresses acting on the tool at high temperature during the cutting process may lead to dislodging of this layer on the tool surface. This diffusion-controlled mechanism is probably behind the decreased wear performance of the WC-Co hardmetal as compared to the wear of TiC-NiMo cermets.

As different from tungsten carbide (WC), titanium carbide (TiC*_x_*) exists as a homogeneous phase over a relatively wide range of carbon content. Dissolution of TiC leads to the formation of substoichiometric carbide particles, not special carbon deficient forms of carbide. As a result, no clear interaction layer at the interface cermet-steel was observed (see Figure 10), i.e., high-temperature diffusion-controlled wear mechanism of TiC-based cermets is different. At high temperatures of the model tests, the iron present in the stainless steel diffuses into the TiC-NiMo cermet tool, forming a solid solution with Ni (see Figure 10). The carbon liberated during the dissolution of TiC dissolves in Ni-based binder, favouring formation of additional metallic carbides. Also, diffusion of carbon to low-carbon austenitic stainless steel takes place during the reaction diffusion tests, favouring formation of the network of chromium carbides in steel (see Figure 9). During the working of stainless steel, this process is similar both to the WC- and TiC- based ceramic-metal composites.

TiC-FeCr cermet demonstrated the highest wear rate (the lowest wear performance) during the model testing. This composite showed also the highest wear gain rate during the increase of the cutting distance (see Figure 7). Diffusion-controlled processes play probably a secondary role in such a rapid degradation process of a cermet tool. The wear performance decrease may be explained by strong chromium steel-to-chromium steel adhesion and tool surface attrition in the contact region of a tool-workpiece.

### 4.3. Working of Copper

Figure 8 and Table 5 demonstrate that WC-Co hardmetals with 90–94 wt.% tungsten carbide outperform all cermets, in particular TiC-NiMo ones which showed the highest degradation resistance during working of the stainless steel (see Figure 7). WC-Co composites show better performance if compared to TiC-NiMo cermets even if they compare unfavourably with hardness (see Table 3 and Figure 5).

While tool wear during the FSW of aluminium and aluminium alloys, steels and titanium and its alloys has attracted interest of several researchers, only a few studies focused on copper alloys are available [10,33,34]. Investigation of the wear mechanism for the H13 steel tool during FSW of Cu-Cr-Zr alloy showed severe wear due to high stresses at elevated temperatures. The sticking of Cu alloy over the tool material surface occurred due to diffusion bonding between copper and steel. The analyses showed that diffusion of Fe from the tool and Cu from the workpiece takes place across the interface. Diffused Fe and Cu form a solid solution layer over the tool surface without any intermetallic formation as per the Fe-Cu phase diagram. High stresses on the tool surface at high temperature may result in dislodging of this layer on the tool surface [33]. Currently, no wear mechanism study is available for hardmetals or cermets during the FSW of copper or copper alloys.

Adhesion as one of the wear mechanisms is confirmed by adherent workpiece (copper) metal deposits on the rake face of tools during the model tests as a result of high stresses acting on the tool at high temperature. In addition to adhesion, diffusion is one of the factors for tool degradation. Figure 11 demonstrates the EDS mapping of the ceramic-metal composites with Co-, Ni- and Fe- based binders in contact with copper after heat treatment at 600 °C. The figure shows a comparatively high diffusion depth of Cu in Ni-alloy of the TiC-NiMo cermet as compared to Cu diffusion in WC-Co and TiC-FeCr composites. It is in agreement with the corresponding phase diagrams Cu-Ni, Cu-Fe and Cu-Co, demonstrating high mutual solubility of Cu and Ni and very low in Cu-Co and Cu-Fe systems even at the temperature of 600 °C. On the other hand, diffusion of Co, Ni and Fe takes place from tools across the interface into the copper workpiece. Intensive diffusion of Cu into the TiC-NiMo binder phase (Cu diffuses faster in Ni than vice-versa) results in a local increase of the binder fraction of the cermet and in a decrease of hardness. It may cause increased wear rate of TiC-NiMo cermets (and also TiC-FeNi cermet) as compared to WC-Co hardmetals and TiC-FeCr cermet. However, more detailed investigations are needed to comment the tool wear mechanism during working of Cu with higher confidence.

## 5. Conclusions

This paper addresses the feasibility of utilizing ceramic-metal composites, in particular TiC-based cermets and WC-Co hardmetals, as tool materials for friction stir welding (FSW) of different metals (aluminium alloy, austenitic stainless steel and copper). Model tests for the wear study of tools from TiC- and WC- based ceramic-metal composites during working of workpiece metals at the cutting temperatures approximately similar to the FSW temperatures and reaction-diffusion tests performed for better understanding of diffusion-controlled processes resulted in the following conclusions.

1. TiC-based cermets with Ni- and Fe- alloy binders show the highest performance in the model tests with workpiece from aluminium alloy.
2. TiC-NiMo cermets demonstrate the highest while cermets with Fe-alloy binders the lowest wear performance in the model tests using counterpart from austenitic stainless steel. An excellent elevated temperature performance is behind the high degradation resistance of TiC-NiMo cermets.
3. WC-Co hardmetals, in particular composites with 90–94 wt.% WC, outperform most of TiC-based cermet grades in the model tests with a workpiece from copper. Hardmetals show better performance if compared to cermets, in particular TiC-NiMo composites demonstrating high wear resistance during working of stainless steel, even if they compare unfavourably with hardness.
4. Irrespective of workpiece metal (aluminium alloy, stainless steel, copper), the most common wear mechanisms of tools from ceramic-metal composites in the model tests are adhesion and diffusion.

## Figures and Tables

**Table 1:** Common tool materials used for FSW of aluminium alloys, copper alloys and stainless steels [15,16].

Welded Material	Aluminium Alloy	Copper and Copper Alloy	Stainless Steel
Tool materials	AISI H13 steel, high carbon steel, HSS steel	AISI H13 steel, tungsten carbide, HSS steel	pcBN, W alloy

**Table 2:** Characteristics of the starting powders.

Material	Chemical Composition (wt.%)	Powder Particle Size (µm)	Supplier
WC	W—base; C—6.13; oth. < 0.14	D50 = 0.86	Wolfram Bergbau und Hütten AG (Sankt Martin im Sulmtal, Austria)
TiC	Ti—base; C_total_—19.12; C_free_—0.15; oth. < 0.31	2.0–3.0	Pacific Particulate Materials Ltd. (New Westminster, BC, Canada)
Co	Co—99.5; oth.—0.5	5.0–6.0	Pacific Particulate Materials Ltd. (New Westminster, BC, Canada)
Fe	Fe—99.72; Si—0.01; P—0.07; Mn—0,02; oth—C	40–90	Rio Tinto (London, UK)
Ni	Ni—99.8; oth.—0.2	3.0–5.0	Pacific Particulate Materials Ltd. (New Westminster, BC, Canada)
Mo	Mo—99.8; oth.—0.2	1.0–3.0	Pacific Particulate Materials Ltd. (New Westminster, BC, Canada)
AISI430L	Fe—base; Fe—16.8; Mn—0.69; Si—0.64; oth < 0.05	10–45	Sandvik Osprey Ltd. (Neath, UK)

**Table 3:** Chemical compositions, average carbide particle size and mechanical properties of tool materials under investigation.

Designation	Initial Composition (wt.%)		Carbide Content after Sintering (vol.%)	Hardness HV30	Fracture Toughness, (MPa∙m^1/2^)	Average Carbide Particle Size, (µm)
	Carbide	Binder				
85WC-Co	85WC	15Co	76.4	1150 ± 20	17.8 ± 0.5	0.91
90WC-Co	90WC	10Co	83.7	1238 ± 6	16.7 ± 0.3	1.19
94WC-Co	94WC	6Co	90.0	1765 ± 25	7.2 ± 0.3	0.48
70TiC-NiMo	70TiC	20Ni; 10Mo	87.7	1340 ± 21	12.6 ± 0.3	1.21
75TiC-NiMo	75TiC	16.7Ni; 8.3Mo	89.9	1403 ± 25	11.4 ± 0.4	1.14
80TiC-NiMo	80TiC	13.3Ni; 6.7Mo	92.1	1492 ± 16	10.1 ± 0.4	1.60
70TiC-FeCr	70TiC	24.9Fe; 5.1Cr	84.0	1352 ± 6	9.1 ± 0.7	1.99
70TiC-FeNi	70TiC	25.8Fe; 4.2Ni	79.3	1379 ± 21	15.2 ± 0.5	1.60

**Table 4:** Chemical composition of counterpart materials for model testing.

Workpiece Material	Chemical Composition (wt%)					
Aluminium alloy (AW6082-T6)	96.9 Al	1.1 Si	0.5 Mn		0.8 Mg	0.7 Other
Copper (Cu-ETP)	99.9 Cu					0.1 Other
Stainless steel (AISI 304)	70.7 Fe	1.6 Mn	18.0 Cr	8.1 Ni	0.3 Si	1.3 Other

**Table 5:** The selection of tool materials for model test.

Workpiece Material	Tool Materials Wear Rate at Maximal Cutting Distances (Shortening of the Cutting Tool Nose Tip, μm)							
	85WC-Co	90WC-Co	94WC-Co	70TiC-NiMo	75TiC-NiMo	80TiC-NiMo	70TiC-FeCr	70TiC-FeNi
Aluminium alloy (AW6082-T6)	7.6	-	fracture	-	3.8	-	6.1	4.8
Stainless steel (AISI 304)	258	174	147	108	42	50	298	191
Copper (Cu-ETP)	28	26	23	37	38	31	27	36
